# Intelligent Wireless Capsule Endoscopy for the Diagnosis of Gastrointestinal Diseases

**DOI:** 10.3390/diagnostics13081445

**Published:** 2023-04-17

**Authors:** Ibrahim M. Mehedi, K. Prahlad Rao, Fahad Mushhabbab Alotaibi, Hadi Mohsen Alkanfery

**Affiliations:** 1 Department of Electrical and Computer Engineering (ECE), King Abdulaziz University, Jeddah 21589, Saudi Arabia; 2 Center of Excellence in Intelligent Engineering Systems (CEIES), King Abdulaziz University, Jeddah 21589, Saudi Arabia

**Keywords:** wireless endoscopy, GI tract, CMOS camera, white light source, data transmission, sphere, capsule, gastrointestinal tract, COMSOL multiphysics, artificial intelligence

## Abstract

Through a wireless capsule endoscope (WCE) fitted with a miniature camera (about an inch), this study aims to examine the role of wireless capsule endoscopy (WCE) in the diagnosis, monitoring, and evaluation of GI (gastrointestinal) disorders. In a wearable belt recorder, a capsule travels through the digestive tract and takes pictures. It attempts to find tiny components that can be used to enhance the WCE. To accomplish this, we followed the steps below: Researching current capsule endoscopy through databases, designing and simulating the device using computers, implanting the system and finding tiny components compatible with capsule size, testing the system and eliminating noise and other problems, and analyzing the results. In the present study, it was shown that a spherical WCE shaper and a smaller WCE with a size of 13.5 diameter, a high resolution, and a high frame rate (8–32 fps) could help patients with pains due to the traditional capsules and provide more accurate pictures as well as prolong the battery life. In addition, the capsule can also be used to reconstruct 3D images. Simulation experiments showed that spherical endoscopic devices are more advantageous than commercial capsule-shaped endoscopic devices for wireless applications. We found that the sphere’s velocity through the fluid was greater than the capsule’s.

## 1. Introduction

As a system of organs that runs from the mouth through the thoracic cavity to reach the abdomen, the gastrointestinal tract (GIT) is responsible for transporting, digesting, and absorption of nutrients in the body of an organism. It works together to accomplish three significant functions. Our lives depend on these functions in which our bodies use accessory organs such as the liver, gallbladder, and pancreas, which perform different functions that collectively allow our bodies to function efficiently. The GIT is the tube that transports food and secretions from mouth to anus after ingested food is slowly pushed through the tract, after which calories and nutrients are absorbed, and finally excreted. Orally ingested drugs, carbohydrates, proteins, fats, minerals, vitamins, and vitamins are all digested in the tract. By chewing, teeth break down starch, which is then converted to maltose by salivary amylases, while the stomach secretes gastric juices containing pepsin that digest proteins to amino acids. The small intestine consists of three parts, namely the duodenum, the jejunum, and the ileum, and they secrete digestive enzymes such as lipase, amylase, maltase, and lactase that break down lipids, amylase, maltose, and lactose, respectively. As the specific place of absorption, the small intestine organs absorb food. The large intestine consists of three anatomical sections: the ascending colon, transverse colon, and descending colon. Reabsorbing water and concentrating feces stored in the rectum are the major functions of the large intestine [1].

Gastrointestinal diseases are defined as disorders affecting GI tracts with different complexity ranges. According to Jones [2], these diseases have severe socioeconomic implications on society with the high cost of investigation and therapy, mostly involving antisecretory drugs, endoscopy, and radiology. Gastroenterologists classify the diseases of the digestive system into the sites of their manifestation: upper bowel diseases and small or lower bowel diseases. Aside from the site-specific classification, they could be grouped into four disease groups: infections, inflammations, malignancy, and autoimmune, specifically peptic ulcer disease, duodenal ulcer disease, Crohn’s disease, and other inflammatory bowel diseases. Disorders of this sort could range from chronic to acute diarrhea, malabsorption, and abdominal pain [3]. Abdominal pain is a typical patient complaint. Therefore, the patients visit a gastroenterology department and often require an examination of the gastric cavity and possibly the small intestine [4]. Intestinal metaplasia and dysplasia are precursors of cancer. Gastric-related complications are the second most deadly neoplasm [5]. In these cases, the early diagnosis and identification of lesions are life-saving procedures that enhance a patient’s survival [6].

Endoscopy and radiology are among the diagnostic equipment, techniques, and procedures used by gastroenterologists to investigate GI complaints [7]. Magnetic resonance enterography (MRE), computed tomography, colonoscopy, push enteroscopy, and small bowel radiology are all diagnostic procedures for looking at the digestive system. Furthermore, diagnostic tools have become more sophisticated with therapeutic overtones that highlight the importance of diagnostic technology for treating certain conditions. There has unfortunately been a possibility of harm from their use. Among the diagnostic techniques used in investigating minor bowel disorders is endoscopy. Traditional endoscopy involves passing a long, flexible tube equipped with a video camera through a patient’s throat or anus or making small abdominal incisions (endoscopy entry). The traditional endoscopic method presents some drawbacks in terms of bleeding, cross-contamination, infections, and perforations. Among the difficulties are apprehensive patients and patients with severe heart and lung disorders or elderly patients [8].

Small intestine images can be obtained noninvasively using MRE. Computers analyze the images created by this method using a magnetic field to create detailed images of organs. This technique involves administering contrast agents/drugs to the organs. Due to its broad view of the small intestine, MRE is useful for diagnosing inflammations, bleeding, and obstructions. Compared to other methods, it takes less than 30 min on the machine and is readily available. Most other diagnostic imaging techniques lack the ability to image thickened walls or stenotic segments. MRE is, however, a complex procedure for patients who have metallic implants such as cochlear implants or cardiac defibrillators due to the interference the implants cause. MRE does not provide images of obscure lesions, as it is not sensitive enough to detect blood loss caused by small, undetectable lesions caused by iron-deficiency anemia [9].

An intravenous contrast material is used in this type of computed tomography imaging. In addition to visualizing abnormalities in the small bowel mucosa and detecting different enteric diseases, CTE provides a good image quality. Aside from its speed, CTE offers painless, accurate, and noninvasive examinations. In addition to exposure to ionizing radiation, the procedure requires intravenous contrast material, which requires exposure to ionizing radiation. Moreover, fibrolytic lesions at stenosis sites are difficult to identify. The small intestine is generally captured in excellent detail with a good overview [10].

A long endoscope is introduced through the mouth and slowly advanced to the small intestine’s proximal areas. A quick visualization of proximal bowels is provided by this technique, which is helpful in viewing obscure GI bleeding or stenosis. Due to its length, it cannot provide images of the distal intestine. Moreover, it is not able to access the entire small intestine due to excessive loop formations of the intestine but still causes discomfort [11]. An important part of diagnosing small intestinal disease is detecting intestinal bleeding with wireless capsule endoscopy, which was launched in 2001. On a capsule (roughly an inch in diameter), a tiny wireless camera is mounted. A wearable belt recorder transmits the pictures taken by the capsule as it travels through the digestive tract. CE can be used efficiently in the small bowel because this region is difficult to evaluate using traditional endoscopy. By using CE, one can see the small intestinal mucosa and diagnose lesions such as ulcers. Due to its ability to visualize the entire small bowel mucosa, CE is very sensitive when diagnosing small bowel pathologies [12].

They are mostly removed by regular bowel movements within 24–48 h; more specifically, they can be used to image the small bowel in cases of Crohn’s disease. The small bowel is commonly affected by Crohn’s disease [13]. In evaluating the disease, the upper and small bowels are considered for optimal diagnostic results. Traditional endoscopy, i.e., push endoscopy, is routinely used in this evaluation. According to Miyoung et al., small bowel follow-up is performed through radiography, which results in different complications in their evaluation methodology. As a result of CE’s advantage in reaching the small bowel and providing a clear, properly contrasted image compared to the other methods [14] that CE has a higher diagnostic yield than ileocolonoscopics (IC), small bowel follow-up (SBTF), and enteroclysis (EC).

There are, however, some complications, although they are minimal in comparison. A common complication of using CE to diagnose GI diseases is capsule retention (CR). It is rare for CE to cause CR. The small bowel, esophagus, and stomach can all be affected. Cancerous lesions, radiation enteritis, stenosis, adhesions, and NSAID usage are associated with an increased risk of CR. It is also a very costly and time-consuming procedure [15]. During transit through the intestine, the capsule’s limited power supply (powered by two coin-cell batteries) may deplete the batteries before the examination is complete. In addition, the capsule’s movement is controlled solely by the asymmetric bowel movement, which may prevent the capsule from examining delicate areas repeatedly [16].

CE offers better advantages including convenient inspection, no trauma or pain, no risk of cross-infection, and no hospitalization despite these complications. Furthermore, the technique does not interfere with patients’ normal activities, and it has proven to be an important advance in diagnosing minor bowel diseases. Aside from being safe and noninvasive, CE outperforms the traditional technique of inserting endoscopic instruments into the body. In using this technique, patients have been spared from pain and risk during gastrointestinal examinations, and it has become their preferred method of examination. Due to its high diagnostic yield, capsule endoscopy has also been used for detecting minor intestinal diseases [17].

Due to the lack of energy storage and the limited volume of the capsule, CE lacks the therapeutic functionality that is commercially available. An exogenous magnetic field can be used to empower and direct the capsule through the GIT [18]. Since its release in 2001, CE’s design has not undergone significant changes. In the literature is available that the capsule shell is made of biocompatible polycarbonate material with two half-spherical ends that are transparent as well as equipped with image LED sensors [19]. In addition to the sensors, there is also an application-specific integrated circuit (ASIC) that is responsible for signal processing and radio transmission. There will be several radio stations allocated to transmit data over a bandwidth of 434.79 MHz as part of the Industrial Scientific and Medical Band. The capsule is powered by batteries that are fitted inside it [19]. 

The commercial size available for CE is less than 32 mm in length and 13 mm in diameter, making it very easy to swallow and move properly through the bowels [20]. Since a single CE camera does not guarantee good results because the orientation inside the bowels is difficult, CE cameras with a variety of cameras have been introduced [21], such as PillCam COLON for colonoscopy and PillCam ESO for esophagus examinations. CE technology is not just limited to the video imaging function but also has the ability to measure the physiology of the intraluminal environment alongside the video imaging function. Measuring GIT parameters such as intraluminal pressure, PH, temperature, and regional transit might assist in the identification of disorders that affect the GIT [22].

As part of the traditional endoscopy procedure, a long, flexible tube equipped with a video camera is passed down the patient’s throat or through the patient’s rectum. The wireless capsule endoscopy (WCE) procedure is the most advanced technology for noninvasive visualization of the gastrointestinal system (GI) that has been developed to date. During WCE, a tiny wireless camera is used that takes pictures of the digestive tract in real time. There is a camera that is placed inside a capsule the size of a vitamin and administered orally. Since the capsule passes through the digestive tract, the camera takes thousands of pictures that are transmitted to a recorder attached to a waist belt on the patient, enabling the doctors to see the small intestine from inside of the body. The capsule will be flushed out of the patient’s body after scanning. There has also been approval for capsule endoscopy to screen the colon for colon polyps as well, which is not possible with conventional colonoscopy. In comparison to traditional endoscopies, the WCE has the major advantage that the patient will not be sedated during the procedure and that the procedure can be performed as an outpatient procedure without having to be admitted to the hospital. In spite of this, there are some technical issues that may limit its use in diagnosis and treatment, such as the battery life, the image resolution, the capsule size, the ability to view the images in real time, the movement of the capsule inside the body, and biocompatibility. As a result, this study is attempting to solve one or more of these problems by modifying the design of the system and installing new components and tools to resolve it. In particular, this study aims to review the role of wireless capsule endoscopy and its efficacy in diagnosing, monitoring, and evaluating disorders of the gastrointestinal tract. The purpose of this study is to review the role of wireless capsule endoscopy and its efficacy in diagnosing, monitoring, and evaluating gastrointestinal disorders since the diagnosis of GI diseases involves a number of technologies that have different capabilities and accompanying complications that can complicate things further. A major objective of the project is to design, improve, and develop a wireless capsule endoscopy that can assist in diagnosing gastrointestinal disorders by focusing on developing tiny and highly specific components that can be used to improve the WCE.

## 2. Methodology

### 2.1. Simulation and Design 

The term laminar flow refers to a flow in which fluid particles are arranged in layers following the Leek method, in which the fluid particles move rapidly between adjacent layers with very little or no compounding and in which the fluid is moving at very low speeds.

Neither cross-currents perpendicular to the flow direction nor eddies or swirls of fluids are present in a fluid flow. On the other hand, the particles of the fluid move excessively in sequence in comparison with particles on the point of the solid surface that follow straight lines parallel to the surface in sequence with the particles of the fluid.

It is characterized by the following:

- High momentum diffusion;
- Low momentum convection.

There are two kinds of flow depending on the fluid’s velocity and viscosity: laminar flow and turbulent flow.

Laminar flow occurs at lower velocities. It reaches a threshold, and then, the flow changes to become turbulent. The speed is set by a dimensionless parameter characterizing the flow, which is referred to as the Reynolds number (RN) and additionally depends on the consistency and density of the fluid and dimensions of the channel.

A laminar flow reactor (LFR) could be a reactor that uses laminar flow to check chemical reactions and method mechanisms.

Turbulent flow could be a less-orderly flow regime characterized by eddies or little packets of fluid particles that end in lateral compounding.

Air curtains are often employed in industrial settings to keep heated or refrigerated air from passing through doorways. 

During the flow of a fluid, the type of flow that occurs affects the amount of heat and mass that is transferred in a fluid system. As a consequence, the dimensionless RN is an essential parameter in the equations that describe whether fully developed flow conditions lead to laminar or turbulent flow conditions. In terms of fluid dynamics, RN refers to the relationship between the inertial force and the viscous force of a fluid, which means how fast the liquid moves in relation to its viscosity regardless of the scale on which the fluid system exists. When a fluid has a slow or very viscous movement, laminar flow will occur as a result of its slow movement. At a certain range of RNs, as the flow shifts from laminar to turbulent at a specific range of RNs, the flow will shift from laminar to turbulent. Low disturbance rates in the fluid or imperfections in the flow system determine the laminar–turbulent transition range in a flow system. When the number of Reynolds number is less than one, the fluid will exhibit Stokes or creeping flow, in which the viscous forces of the fluid dominate the inertial forces of the fluid.

In the same way that laminar flow occurs when there is a low Reynolds number in pipes, tubes, and wind tunnels, turbulent flow and its phenomena such as vortex-induced vibration and vortex shedding occur when the Reynolds number is high.

Computational fluid dynamics (CFD) is a mathematical software application that provides precision, reliability, flexibility, and a wide range of application options. As a result of CFD, it is possible to improve the designs without having to create accurate models such as those from a wind tunnel, for example. Additionally, it may be able to provide accurate quantitative predictions of fluid interactions and trade-offs as well.

Traditionally, CFD is used by engineers and scientists to understand, predict, and design fluid flow in various types of systems (open/closed). As a result of these simulations, new and better products are developed as well as improvements in various processes and devices that are reliable. 

Simulations can give accurate estimates to the following:

(1) Flow patterns;
(2) Pressure losses;
(3) Forces on submerged objects;
(4) Temperature distributions;
(5) Variations in fluid composition within a system.

It is important to note that the general capabilities of the CFD module encompass modeling stationary and time-dependent fluid flow issues in both two- and three-dimensional areas. A large variety of flow formulations are defined in several fluid flow interfaces, which are used to line up and solve the spread of fluid flow issues. These physics interfaces demonstrate how a fluid flow problem can be modeled using physical quantities such as velocity and pressure as well as physical properties such as viscosity. A variety of fluid flow interfaces have been found to be suitable for a variety of different flows including laminar and turbulent single-phase, multiphase, non-isothermal, and reacting flows. In order to resolve these equations, finite element formulations are used for fluid flow and time-dependent problems based on the time factor and time-dependent solver algorithms as well as the traditional global methods, also known as damped Newton methods, to solve the equations. In the CFD module, the workflow is sorted in a way that is easy to follow and is clearly defined by the following steps: (1) define the geometry, (2) select the fluid to be modeled, (3) select the flow type, (2) outline the boundary and initial conditions, (3) outline the finite element mesh, (6) select a solver, and (7) visualize the results. All of these steps are taken by the COMSOL software. As a first step, this software creates geometry and then adds boundary conditions on top of that. The mesh will then be generated after that. The final step is to solve the steps one by one. It is also automatically carried out in the last step using default settings that are tuned for each specific fluid flow interface. There is an application library in the CFD module that describes the fluid Flow interfaces and their completely different options through tutorials and benchmark examples for the different types of flows. The purpose of this website is to provide users with a variety of models of industrial equipment and devices, tutorials for practice, and benchmark applications for verifying and validating the fluid flow interfaces.

**Figure 1:**
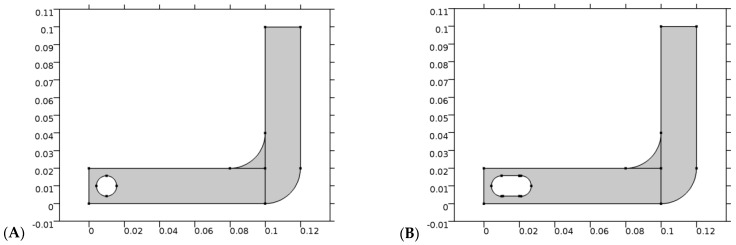
A simulation of a segmented GI tract and the motion of the endoscopic modules along that tract. (**A**) Spherical-shaped modules; (**B**) capsule-shaped modules.

As part of this experiment, a hollow tube filled with water was created, simulating the GI tract. It is important to note that water is considered the medium inside the tube because it is the major component of the fluid that is present in the GI tract. In the second step, a capsule and a spherical object are simulated in 2D separately, passing through individual tubes as they pass through the capsule. Figure 1 shows the geometry of the model that was created by the software that was used to create the model. 

**Figure 2:**
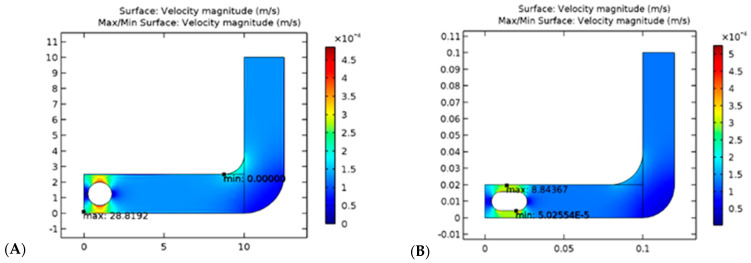
Comparison of velocity for spherical (**A**) and capsule (**B**) geometries in the GI tract.

It has been possible to solve the models of capsules and spherical objects. As a result of considering a laminar fluid flow pattern through a tube, the solution was based on CFD principles. According to the results presented in Figure 2, the velocity of the sphere through the fluid is greater than the velocity of the capsule shape through the same fluid, which means the spherical-shaped module excretes more rapidly than the bowel and is more likely to exit easily from the bowel following completion of the examination. According to the fundamentals of engineering mechanics, velocity and friction are inversely proportional to one another. As the velocity increases, the friction will decrease as well. In comparison to the capsule, the spherical-shaped geometry will result in a decrease in friction on the lateral wall of the gastrointestinal tract due to the increased velocity. In addition, the spherical shape has a smaller outer surface area than the capsule, which allows the smooth sphere to roll more freely in the fluid-filled path, thereby reducing the amount of friction created during rolling. As can be seen from the table below, it is noted that rolling friction is lower than sliding friction, and sliding friction is lower than static friction.

**Figure 3:**
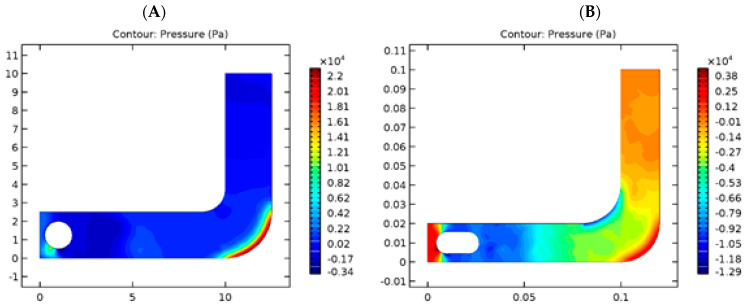
Comparison of pressure for spherical (**A**) and capsule (**B**) geometries in the GI tract.

The pressure exerted on each shape was additionally analyzed. Figure 3 shows pressure values on spherical and capsule geometries within the tube considered. Based on the simulation results, it was found that the sphere form has lower pressure, thanks to its pure mathematics advantage, as compared to the capsule, which has a higher area. Thus, there will be more significant pressure on the laminar flow, and the capsule form will have additional turbulent flow compared with the sphere form, which could produce structural injury on the inner wall of the digestive tube.

### 2.2. Electronic Circuit Simulation

As we know, electronic circuit simulators are electronic programs that allow us to capture schematics, simulate electronic circuits, and design electronic circuits. They are currently being used for a variety of purposes including studying the efficiency of equipment circuits, power consumption, distributed power in circuits, operational efficiency, and identifying devices with short shelf lives.

There are a few programs that simulate electronic circuits, but the three most well-known ones are Multisim, Lab VIEW, and Proteus. An electronic circuit schematic can significantly improve the design efficiency of an electronic circuit by spotting faulty designs known in advance and inserting the correct ones into the behavior of an electronic circuit before it is built.

Through the use of the Multisim program, the WCE project has followed the process of circuit design. As a result, it has a large library of electronic components such as resistors, fuses, capacitors, photodiodes, transistors, integrated circuits, and microcontroller-integrated circuits. Furthermore, users are also able to define components that allow them to generate specific components that they desire. Additionally, the Multisim program has a variety of measuring tools such as a Multimeter function generator, a Wattmeter, a power supply, an oscilloscope, a logic analyzer, and many others.

When designing a WCE board, the first step is to simulate the electronic circuit that aims to calculate the resistance of the LED, the value of the crystal oscillator, the value of the decoupling capacitor, the power distribution of the circuit, and the reading of the output CMOS camera sensor. Moreover, it determines the amount of power consumed by the circuit, which helps us to know how long the battery will last. In order to ensure that the circuit worked appropriately without errors, we designed and simulated our experiment. In addition, we also used it to study the power consumption of the wireless capsule endoscope board as well as the current consumption, resistance value, and voltage distribution of the board.

### 2.3. Schematic Editor

**Figure 4:**
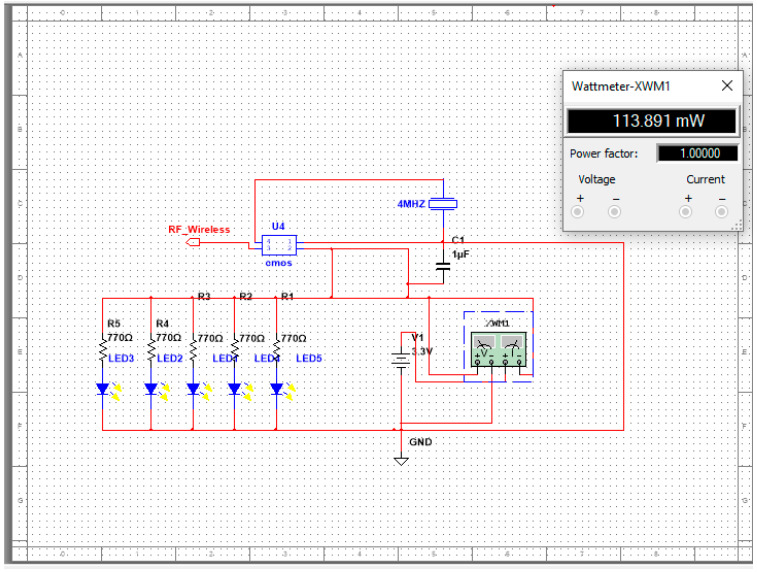
Circuit of 5-LED capsule.

**Figure 5:**
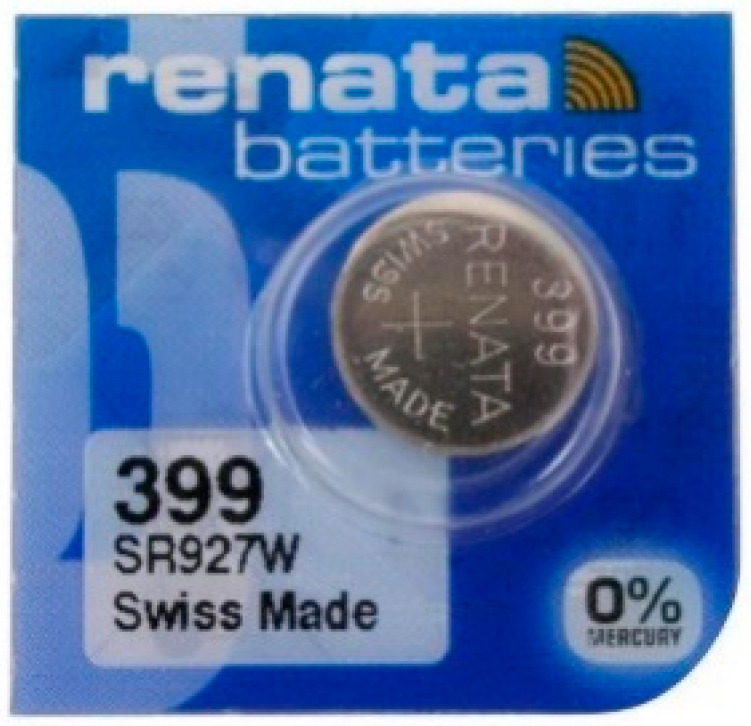
399 Renata battery.

**Figure 6:**
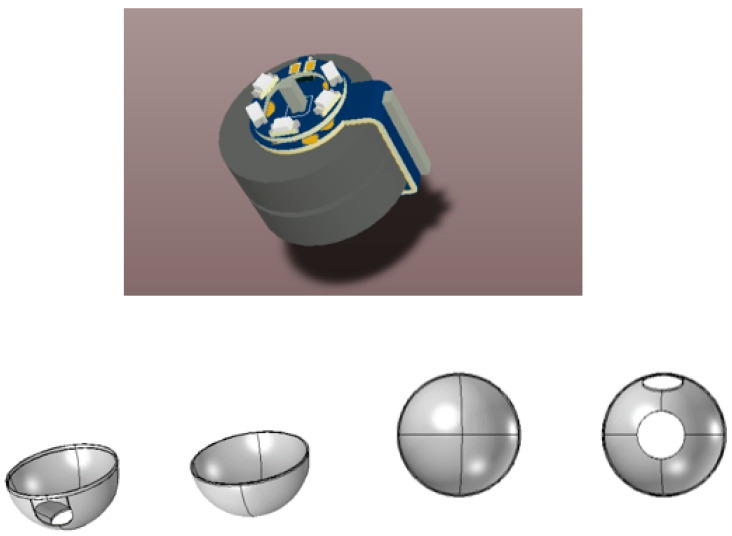
Three-dimensional capsule and outer cover.

The final design of the electronic schematic editor was completed, and all components work with high performance. The simulation succeeded, as shown in the figure; the components are designed correctly, power consumption is measured, and the power and voltage of components are distributed proficiently. For example, Figure 4 and Figure 5 shows the schematic of the LED circuit, and the wattage is 113.8 mW; Figure 6 shows the schematic of the LED circuit, and the wattage is 100.2 mW.

### 2.4. PCB Layout Design

In recent years, the printed circuit board (PCB) industry has grown significantly and has become more accurate and sensitive to its customers’ needs. In the past, this device was quite large and had one or two layers with a significant number of electronic components. Today, it has more than seven layers, and the size of the electronic components has been reduced to a point where it is no longer possible to design them manually. In order to find a design through a method that is more accurate and more efficient in terms of accuracy and efficiency than the manual or traditional method, PCB layout design software has been developed. PCB layout design software is a 3D designer for a printed circuit board used to design the 3D shape and PCB layer with an international standard requirement so that all design requirements can be met to generate the grabber file of the PCB design.

A Gerber file is a file that almost contains all the layers of a PCB, such as the layer in the physical board, the layer that covers the component, the top signal layer, the bottom signal layer, the solder masking layers, and so on.

There are some PCB layout designs that provide administration functions such as checking the warehouse component store, the bill of materials (BOM), suggestions for similar electronic components, and ordering components from the warehouse. A number of PCB layout software programs are available on the market, such as Altium Designer and Eagle PCB Design Software. With the help of Altium Designer software, we designed our WCE board in this study, allowing us to transfer the circuit design from concept to schematic to layout to a final product in a seamless manner. In addition, Altium Designer provided data management tools, ECAD libraries, and support for innovative rigid-flex PCB designs.

### 2.5. RF Wireless and Video Wire 

It is important to note that WCE’s RF wireless technique is complex despite the fact that it is an integral part of the operation. There is a need for RF to meet the performance and size requirements for implantable communication systems with the use of Medical Implantable Communication Service bands (MICS) such as ultra-low power (ULP). There are many applications that use the RF of MICS such as wireless capsule endoscopes, pacemakers, implantable cardioverter defibrillators (ICDs), neurostimulators, implantable sensors and diagnostic devices, and drug pumps. As a result of this, we designed a separate board for the RF transceiver in the PCB design. It should be noted that the RF board should contain the ULP RF transmitter IC and the antenna, along with several passive components such as a resistor, capacitor, etc.

In order to transmit data through the human body, MICS, a transmission system that operates at a frequency of 402–405 MHz, is suitable. As a result, its vendor (Microsemi Semiconductor & System Solutions, which produces the ULP MICS RF) sells a transceiver at a low data rate (effective data rate of around 0.5 Mbps [23]), which is not sufficient even for an endoscopic capsule application.

The silver oxide-ion batteries that are used in implantable biomedical devices play a vital role in ensuring the success of their deployment and their ability to treat human diseases. The purpose of these devices is to provide therapy on a predetermined schedule to the patient and to check his or her condition.

Several devices have been developed for the treatment of a variety of human health problems over the years. Depending on the type of device, diagnosis, or therapy, the battery’s functional requirements may vary. A silver oxide battery can be used to manufacture a wide range of medical implant applications (pack makers, drug delivery systems, etc.) as well as many other applications in the field of medicine. The batteries provide services to minimize the surgical frequency, maintain safety during installation and use, ensure predictable performance that can be interrogated to provide state-of-discharge information, and be highly reliable.

The batteries must have volumetric and high energy density to enable the design of small devices that minimize discomfort for the patient. Additionally, long-term stability, high-quality performance, and high volumetric energy density are critical characteristics of the success of biomedical implants.

The wireless camera endoscope is powered by two coin batteries, namely 1.55 V silver oxide-ion number 399 from Renata battery company, as shown in Figure 5. This battery has a size of 9.5 mm × 2.6 mm, which makes it small; each coin battery has a 45 mAh rating; thus, the total rating is 90 mAh. Additionally, in the primitive calculation, the design also contains a CMOS camera with 25 mW and 3.1 V, which equals 7.5 mAh, which is considered low consumption. In addition, the design is ultra-low power (ULP). As a result, the electronic circuit simulation result will show the power consumption calculation.

Using an intelligent power-management system for the data recorder, some WCE researchers have managed to save energy for more than 12 h by regulating the image-transmission rate and applying a sleep mode to the capsule. Recorders can recognize the capsule’s location inside the digestive tube and adjust transmission rates accordingly. The capturing of images begins after half an hour. In the stomach, the recorder recognizes the capsule and transmits six images per minute at a slow rate. In order to increase the transmission rate, the recorder detects the capsule entering the small intestine. Capsule movement is also detected by the recorder. The camera captures up to 35 images per second if the capsule moves. When the capsule resides in the stomach for over an hour, the recorder can make a sound signal and a vibration to ingest a prokinetic agent. Its revolutionary design and technological developments are awe-inspiring. The diagnostic yield of WCE in the colon cannot be compared to that of colonoscopy. This method, however, cannot be used in this study because it only transmits images. This study may provide new insights into energy development and reducing energy consumption. A reed then switches a vital device: a magnetic switch that controls electricity flow. An envelope-shaped glass tube contains two or three contact pin reeds. In an electric circuit, a reed switch acts as a bridge or gate, passing electricity when two reeds are in contact. Magnetic or electromagnetic fields control the magnetic switches.

As a durable component, it can be used for medical devices that could continue to function for millions of operations without the need to be replaced, such as pacemakers and neurostimulators. The other advantage of this device is that it can operate without the need for electrical power for operation, which makes it extremely useful in battery-operated devices including wireless capsule endoscopes and in-canal hearing aids, for example. The reed switches are used in wireless capsule endoscope that saves energy. The reed switches are classified into two main types:

- A standard closed reed switch will stop the circuit with a magnetic field;
- A standard open reed switch will run on the circuit with a magnetic field.

A WCE factory usually uses the standard close reed switch and tests the capsule, then puts it in a carton box, and attaches the magnetic piece to a carton.

The capsule will automatically work when it is taken out of the box. On the other hand, the time elapsed in travel from the factory to the patient may be more than a month. However, the lifetime of a battery is shorter than this long duration.

We used the reed key to solve this problem by using in our WCE project a switch reed, spst-no 100 ma, 24 maximum volt, and 0.071” Dia × 0.276” L (1.80 mm × 7.00 mm) size. 

### 2.6. Capsule Component and Outer Cover 

The components of capsule system are divided into the CMOS camera, lens, battery, LED light, reed switch, and outer cover. The others included external components such as the recorder unit and computer workstation. The 3D shape of PCB was designed through Altium designer software in Figure 6. The design contains the following electronic internal components:

- A CMOS camera;
- Lens of the CMOS camera;
- Five light sources;
- Two coin batteries;
- Radio transmitter;
- Reed switch.

**Figure 7:**
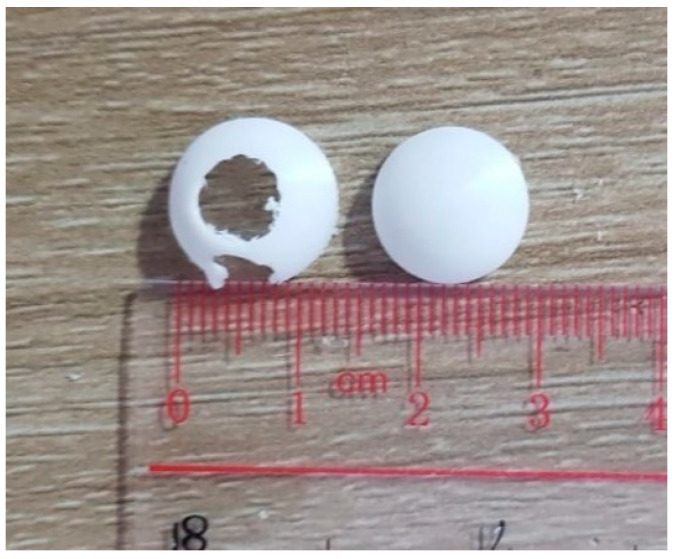
Capsule cover after 3D printing.

STL (stereolithography), as shown in Figure 7, was generated for the outer cover by COMOSL Multiphysics, and then, the outer cover was printed through a 3D printer. PLA plastic material was used as well. The outer cover should be biocompatible in stomach and GI environments. We did not use humans and animals in the experiment.

As shown in Figure 7, the outer capsule was covered after 3D printing. 

### 2.7. External Component 

Recorder unit

**Figure 8:**
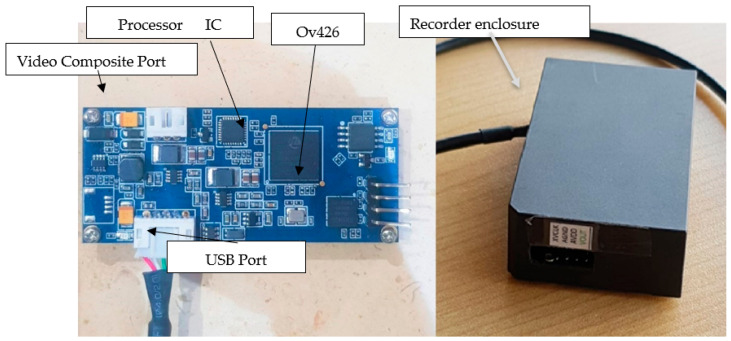
Recorder unit.

The recorder unit, i.e., the decoder module or data logger, is fundamental in the experiment because it improves our results. The recorder unit, as shown in Figure 8, is described and has two operation missions.

First, it converts the analog video signal to digital.

As a result, it converts the output of our CMOS camera, a RAW analog video file, into a digital file. The ICS of our recording unit, known as the OV426 (OMNIVISION, Santa Clara, CA, USA), is a single-chip solution designed for a small medical image camera such as the OV6946 (OMNIVISION, Santa Clara, CA, USA). Using a built-in A/D converter (ADC), a black-level calibration (BLC), AEC/AGC, and a final digital video parallel output (DVP), this unit provides an integrated analog-to-digital data-conversion system. It is also important to note that the OV426 has a standard SCCB interface for communicating with the system and manipulating the functions that have already been mentioned. The device has other functions as well, including the ability to convert a raw video, allow the signal from the sensor camera to be encoded into (ten bits) so it can be transferred to a pal port or any other type of video, and then transfer the video data to the computer.

Next, it processes and communicates with other hardware devices.

In order to communicate with other hardware devices such as computers, the Internet, and printers, the processor’s main purpose is to store the information in flash memory, display it, and process images as needed, for example, by removing noise, controlling the frame rate of the camera, and using the serial peripheral interface (SPI) with a CMOS camera.

It has been reported that most data loggers discussed in the literature have a wired connection to biosensors. The wireless link as well as the graphical display unit are not able to be utilized efficiently for logging data from wireless capsule endoscopy because of the lack of adequate storage capacity. This means that there is a great opportunity for research on designing data loggers that can be used for capsule endoscopy, which would have all the resources needed for such an application to be successful.

### 2.8. Workstation PC

**Figure 9:**
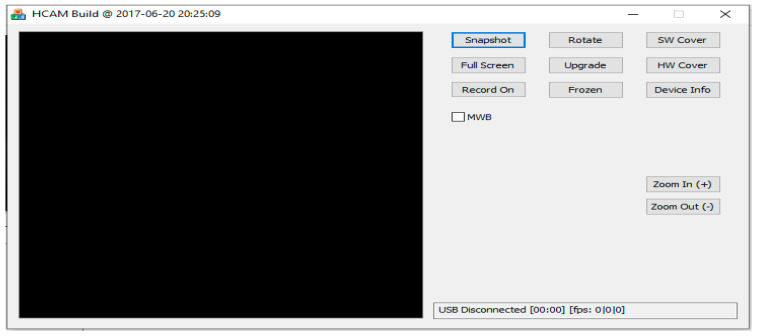
PC software.

As soon as the patient images were examined, they were transferred to a PC, decoded, and displayed on a workstation computer after they were transmitted. As seen in Figure 9, the computer can perform many different research missions such as previewing images in a special software program as shown in Figure 9, processing images, filtering noise, learning machine learning, visualizing images in three dimensions, or using a workstation computer for performing office work such as sending emails, printing images, writing results, and so forth.

## 3. Implementation

### 3.1. The Implementation of a Wireless Capsule Endoscope Has Five Steps

The steps are as follows:

- Fabricate the PCB through the CNC drill:

The CNC drill machine was used to drill and fabricate the PCB.

- Check the PCB pad and track the transparencies sheet:

A transparency sheet is a printed sheet from the transparency layer that checks the PCB conductive tracks and pad position.

- Implement the solder mask:

A solder mask is a thin layer of polymer applied to the copper tracks of a printed circuit board (PCB) for protection from oxidation and to prevent solder bridges from forming between closely spaced solder pads.

The non-solder mask defined (NSMD) technique was used in WCE because it contains BGA components.

**Figure 10:**
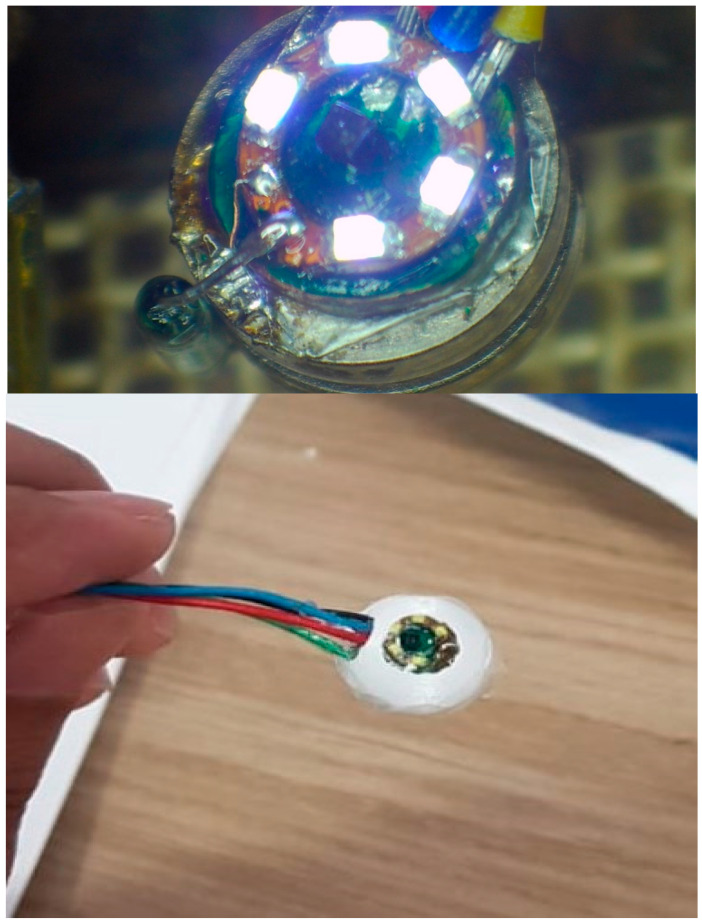
Image after installing all components and before and after the outer cover.

- Fix the component:

All components were fixed on the binocular microscope stage and used heated air to solder the components. After fixed, the component was checked to solder, and the components pin was contacted, as shown in Figure 10. 

### 3.2. Images Processing and Calibration 

#### 3.2.1. Noise Remove

It is important to keep in mind that image noise appears in images as pixels and has fewer light fluctuations to report in the intended image, but it is over-amplified by boosted ISO values. Aside from exposure, images are also susceptible to a number of other issues that can result in noise being generated in the final image as well. Noise can also be caused by the heat generated by the sensor itself. As far as the noise is concerned, it can be divided into two main categories: internal noise and interference noise. In other words, internal noise is the noise that is created by the systems without interference from external sources. There are several types of internal noise that can be identified, such as Gaussian noise, fixed-pattern noise, salt and pepper noise, shot and darkness noise, quantization noise, and anisotropic noise. When a recorded signal is interfered with by a number of natural or man-made signals, it is known as interference or periodic noise. In most cases, it appears as a fixed pattern overlayer over the desired image. In the course of studying the CMOS camera of WCE, we found periodic noise as a result of the harness’s high impedance, which caused periodic noise to occur. As a first step, we reduced the impedance of the harness by using shielded cable, good soldering, and a good electronic connector. The first step to start the design and simulate the circuit generated a specific component for CMOS through our CMOS Camera Ovm6946, in which the specific component was created and added to Multisim library program. We obtained the information of the CMOS camera from the datasheet (see Table 1).

**Figure 11:**
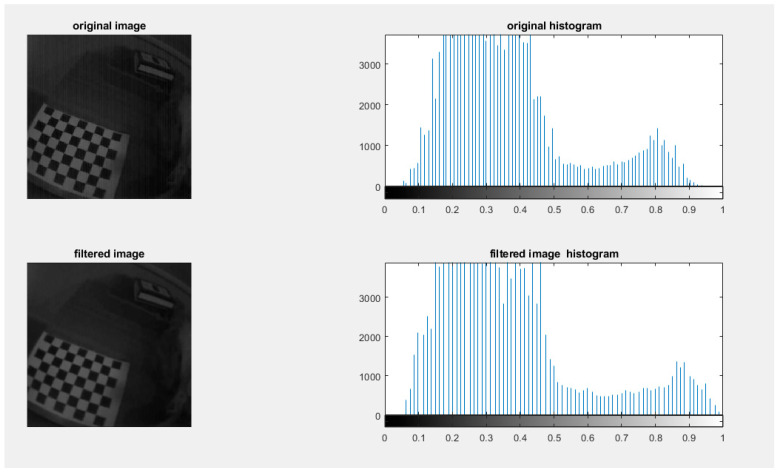
Comparison between the original image and filtered Image.

In the second step, the rest of the noise was removed using a computer-aided assistant that was created by a mathematical algorithm. As part of this process, we used MATLAB software to complete this mission, which allowed us to code the process of removing and analyzing the noise using MATLAB. It is possible to remove noise from images by using the F special (disk) filter. The F special(disk) is defined by MATLAB by creating a two-dimensional filter of the specified type using the two-dimensional filter h. There are some filter types that have additional parameters, as shown in the following syntaxes. In order to use a filter with F special returns, one needs to have a correlation kernel, which is the appropriate form. There is a comparison between the original image and the filtered image, which can be seen in the image below. As shown in Figure 11, the image filter in the histogram graph is smoother than the original, and the vertical pattern noise is removed.

#### 3.2.2. Camera Calibration

**Figure 12:**
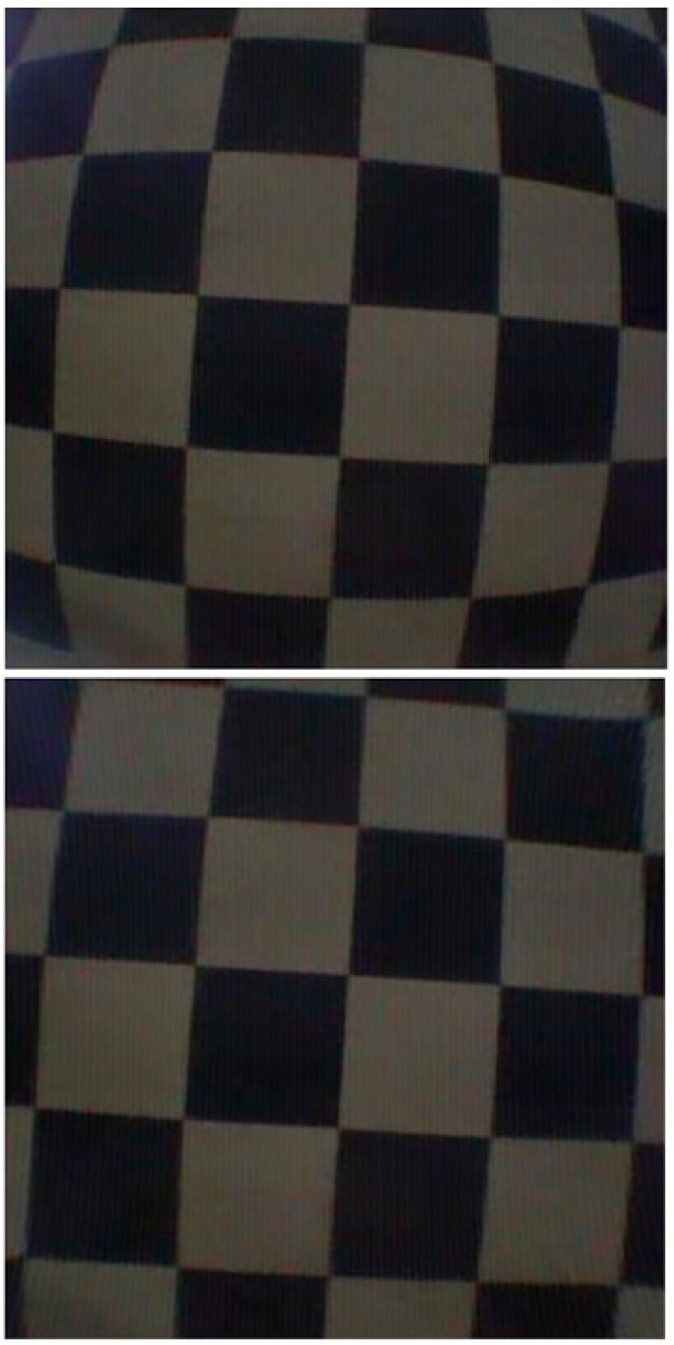
The original image and image after camera calibration.

Calibrating a camera estimates intrinsic and extrinsic parameters. The camera’s intrinsic parameters include skew, distortion, focal length, and image center. A scene’s intrinsic parameters allow the first step of 3D computer vision to estimate the scene’s structure in Euclidean space and remove lens distortion. Extrinsic parameters describe the transformation between the camera and its external world, also called camera pose or external parameters. The calibration board has 63 squares (32 black and 31 white). The method detects lens distortion. Our experiment calibrated the calibration board with 42 pictures from the WCE camera (more pictures increase calibration accuracy). In the next step, we generated MATLAB code to estimate intrinsic and extrinsic parameters from WCE camera images; then, the code was calibrated. The original image and the calibrated image are shown in Figure 12.

White balance (WB) is the process of removing incorrect color casts that affect the colors of the image; the white balance is necessary for the camera and protects the image from the saturation of light and affects. The unwanted color refers to the coolness of white light or a relative warmth. 

## 4. Results

### 4.1. Simulation and Results

#### 4.1.1. Comparison between Shape Results

**Figure 13:**
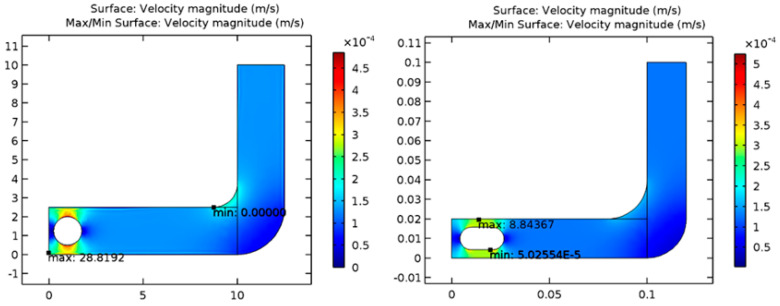
Comparison of velocity for the GI tract’s spherical (**Left**) and capsule (**Right**) geometries.

Computational fluid dynamics (CFD) was used to solve models of capsules and spheres using laminar flow patterns through tubes. As shown in Figure 13, the results showed that the velocity of the sphere through the fluid is more effective than that of the capsule shape in the same fluid. In other words, the spherical-shaped module excretes faster from the bowel, enabling easy exit. According to engineering mechanics fundamentals, velocity and friction are inversely proportional; as velocity increases, friction decreases. In addition, spherical-shaped geometry reduces friction on the lateral wall of the GI tract when velocity increases. Further, the sphere has a smaller outer surface area than the capsule, which makes it easier for the smooth sphere to roll in the fluid-filled tract. As a final point, it is worth noting that the rolling friction is less than the sliding friction, while the sliding friction is lower than the static friction.

**Figure 14:**
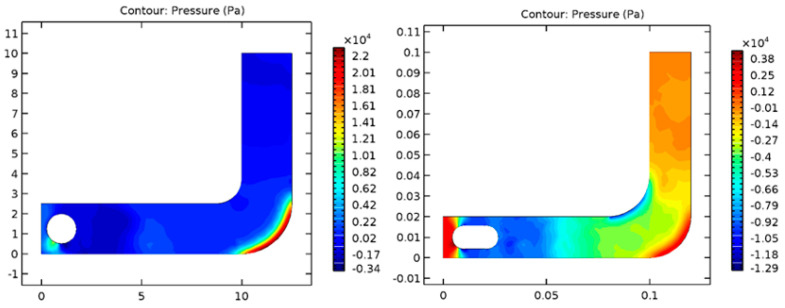
Comparison of pressure for spherical (**Left**) and capsule (**Right**) geometries in the GI tract.

We additionally analyzed the pressure exerted on both shapes. Figure 14 shows the pressure values on spherical and capsule geometries. Based on simulation results, the spherical has less pressure due to its geometric advantage over the capsule, which has a larger surface area [24]. The capsule shape has a more turbulent flow than the sphere shape due to the robust pressure in laminar flow. 

By Multisim software and the electronic diagram of WCE, the wireless capsule endoscope of the electronic circuit was simulated, and we determined the value of the electronic component. As well as the electronic simulation for the power consumption, the power consumption of the system was calculated as well. This simulation result shows that the power consumption of the circuit with 5 LED lights is 113 mW, while it is 100 mW with 3 LED lights, which is sufficient, indicating that increasing the number of LED lights may cause the image to become overly saturated and bright. It is also important to take into account that the simulation calculated a power consumption of WCE, which is 100 mW, and the capacity of two coin batteries is 190 Watts, which means the battery life will last around 2 h. Alternatively, if the camera is running continuously with 32 frames per second, it will take more than 8 h, but if it is run intermittently, it will only take eight frames per second.

**Figure 15:**
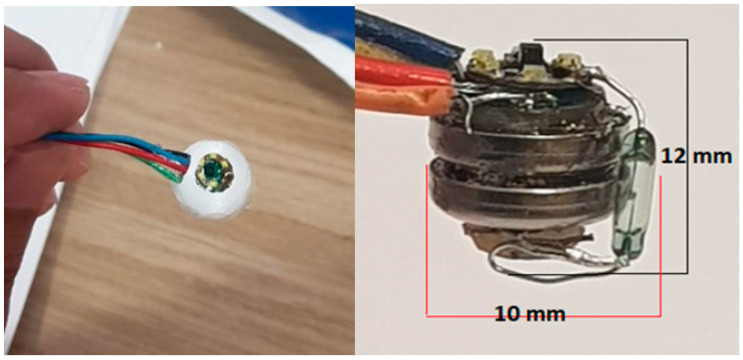
The final shape of the capsule and the capsule core.

As can be seen in Figure 15, the design was manufactured by assembling two circular PCBs together. According to the figures, each one of them has a 6 mm diameter attached to dual batteries with a total capacity of 9.2 mm, as shown in the figures. In a high-resolution microscope of electronic work, hot-air soldering fixed the PCBs with a CNC drill.

A third party was contacted to design, manufacture, and assemble the two PCBs that were provided by the third party. In addition to this, they were stacked on top of other board-to-board connectors that were used. In this project, the PCB capsules developed by the team were packed inside a spherical (6.5 mm in diameter) casing. It is estimated that the weight of the prototype, including the casing, the PCBs, and the two coins, is 5 g. Due to the fact that the fabricated RF chip will not be sold individually, the prototypes will not contain the fabricated RF chip. With the development of the system over a period of time, a flexible and programmable FPGA was developed on a platform that enables the design to be updated without having to undergo the lengthy fabrication process of an ASIC over a period of time.

**Figure 16:**
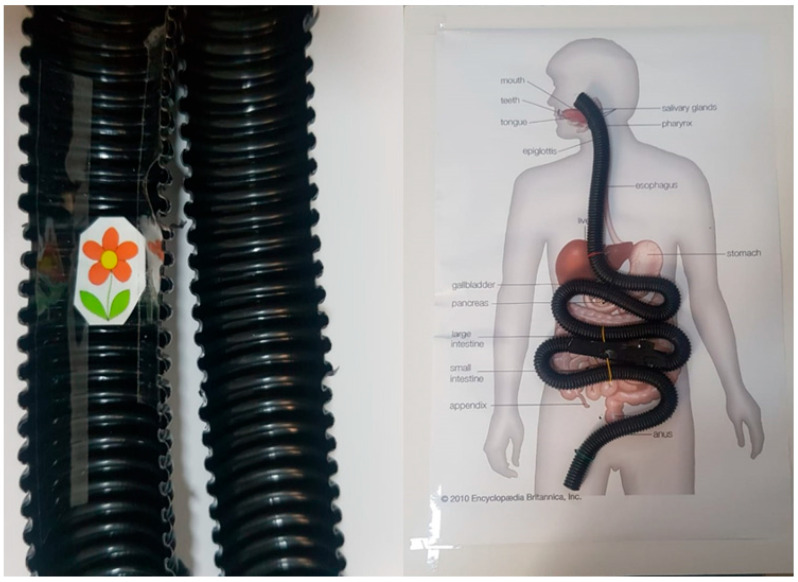
Tube with flower sample and the entire module test.

**Figure 17:**
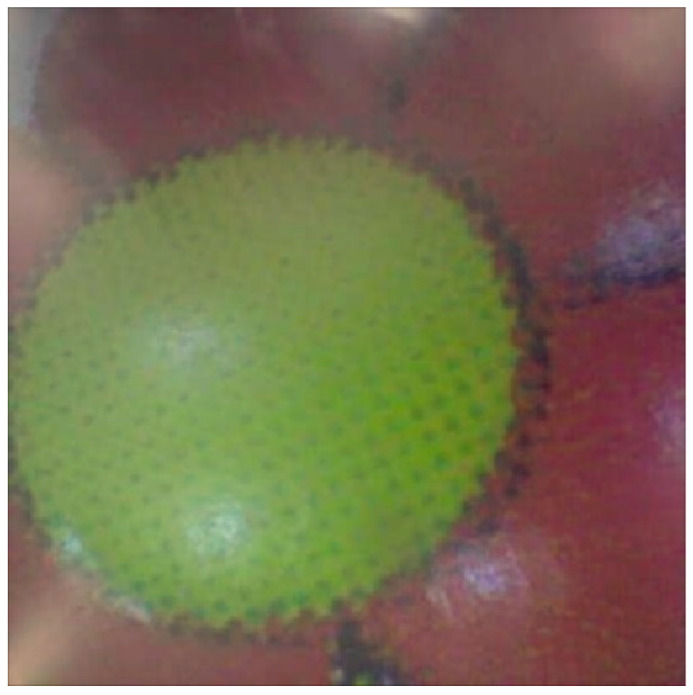
Image capture from WCE.

Figure 16 shows a comparison between our capsule and a child’s small intestine; our capsule is smaller than a child’s small intestine, and its diameter is 13.50 mm. Figure 17 shows a comparison between our capsule and a child’s toy; our capsule is smaller than the child’s toy. The size of the WCE core without cover is 10 × 12 mm, and it can be cut into any shape that is needed. MATLAB code was used to determine the resolution performance of our WCE, which is based on the picture forms of our WCE as well as the horizontal and vertical resolutions, which are 96 dots per inch (dots per inch). There are 400 pixels by 400 pixels in the image. There is a depth of 24 bits in the output image. As shown in Table 1, the focal length of the lens is 35 mm.

#### 4.1.2. Module Test

The module test was designed through a tube with an inner diameter of 2.5 cm because it is the same size as the small intestine. The tube was fixed on a plastic board, and the image of the GI inside the tube, denoted by the small flower, is shown in Figure 16. As shown in Figure 17, the images were very clear and had high resolution.

## 5. Discussion

It has been proven that the capsule endoscopy (CE) can significantly affect the capsule’s lifetime, image resolution, and viewing angle in individual medical products. In spite of that, more research is still required to identify diseases, obtain high-quality images, and extend the lifespan of capsules. The capsule endoscopy is, as we mentioned earlier, a unique technology that allows for highly effective and noninvasive imaging of the gastrointestinal tract through the use of capsules. The capsule endoscopy has gained a significant role in both the diagnosis and monitoring of Crohn’s disease and celiac disease as well as in the surveillance of small bowel tumors and polyps in selected patients [25]. Research on the use of CE in various diseases that affect the gastrointestinal tube is rapidly accumulating in the literature, so there is a need to continuously improve these tools across a broad range of diseases that come into contact with the gastrointestinal tube. 

The capsule in this study was compared and contrasted with other types of capsules that have the same function as the capsule of this study, as shown in Table 1. We began with the size of the capsule. In general, the size of the capsule and its shape are very important factors when it comes to its effectiveness. If the capsule is to be swallowed, it should have a smaller size and a more streamlined shape that allows it to easily move from the time it is swallowed until it is expelled by feces from the intestine. The ease of movement of the capsule may reduce the possibility of intestinal blockage by the capsule and may also reduce the pain of swallowing the capsule. However, there is a possibility that a spherical shape with a diameter of 14 mm, the largest diameter among all other spherical shapes, could be the best size and shape to be more streamlined.

In terms of the battery life, capsules have the same capacity, but with our design, it is possible to extend the battery life to more than 8 h, but it is affected by the number of frames per second. Our design for WCE has a resolution of 400 × 400, which is suitable for the field of view and is enough to clearly see the image of the flower, as shown in Figure 17. Additionally, there is no distortion noise due to the lens [26]. 

It is reported in the literature review that the primary limitation of using the WCE is the low frame rate, which has a detrimental effect on image resolution and limits its wider application. We increased the frame rate to (8–32 frames per second), which enhances the quality of the image as well as opens up a new field for 3D image reconstruction [26].

In this study, the field of view of the capsule is 120 degrees, which is adequate when compared to other capsules that have a larger field of view than our capsule because they make a longer field of view, which reduces the resolution of the image. There is also the problem of more processing and lens distortion, which leads to a great deal of difficulty in removing and affecting the captured images as a result of the distortion. As a final note, it is important to note that this design differs from other designs because the communication is provided through the use of a wireless device. There is an area on the PCB for a wireless component, but the manufacturer of the wireless component does not sell wireless capsules for personal use. An endoscopy capsule is a pill-shaped wireless device with a slippery coating that makes it easy to swallow, and it measures 13.5 mm in diameter. A white-blue light-emitting diode is used as the light source, and a lens, an imaging chip, batteries, and a radio transmitter with an internal antenna are also used. A 400 × 400 pixel image size results. As soon as the patient swallows the capsule, it moves through the intestine via peristalsis and is excreted in the stool as a result. The camera can take up to two images per second as it sweeps the intestine and transmits these to eight lead sensor arrays, arranged in a specific manner and taped to the anterior abdominal wall, transmitting these images to the cameras, which are connected to a recording device in the belt for the duration of the battery life, which is 2 h for full frame rate and 8 h for the eight-frame per second rate.

For the development of wireless capsule endoscopy systems to be effective, it is essential to study the shape of the human GI tract or digestive tract in order to make informed decisions regarding its appropriate development. There are four main parts of the human gastrointestinal tract that are shown in Figure 16: the esophagus, the stomach, the small intestine or bowel, and the large intestine or colon. There are approximately eight meters of digestive tract in an adult, with the small intestine occupying approximately five to six meters of the entire digestive tract, which is approximately 3–4 cm in diameter.

Compared with traditional endoscopy, capsule endoscopy has a high sensitivity in terms of identifying abnormalities within the GI tract, and it also provides a visual of the entire length of the small intestine during the endoscopy. In spite of this, the capsule endoscopes still do not provide high image resolutions and frame rates when it comes to images. In order to boost the data rate with optimized power consumption, this study generated a highly efficient transceiver chip. According to our results, with the help of a transceiver, the battery life was increased to 8 h. It is worth mentioning that one of the most concerning disadvantages is the limited control over the movement of the capsules. As the movement of the capsules is dependent on the natural transit of the bowel, the system is not capable of repeating the examination of the same location on a regular basis. The asymmetrical bowel transit (slow or fast) increases the likelihood that important information might not be detected. In addition, all the capsules were powered by two coin-cell batteries of 60 Watts. 

## 6. Conclusions 

This study evaluated a non-invasive method of evaluating the small intestine and gastrointestinal tract that makes use of wireless capsule endoscopy. It has been proven that it is an effective tool for treating many medical conditions in the past. This technology, however, has some limitations, such as a low-resolution images, a short battery life, and an increased risk of blockage in the intestine if the battery is not effectively charged. A wireless capsule endoscope model was developed with high specifications and unique features in terms of the shape and size of the capsule, the resolution, and the speed of frames of the endoscope in this study.

As part of the study, three main steps were followed: first, we performed a simulation with computer assistance in making the right decisions, such as comparing capsule shapes, simulating electronic circuits and selecting the correct electronic components, and designing the 3D PCB and PCB layout. Secondly, the PCB was fabricated by using a special NSMD solder mask technique that fit the BGA component on it, followed by the fabrication of the PCB. In addition, the manual introduces the solder method and includes the necessary parts to be able to operate the WCE.

By removing the noises from the image and processing it, the third step was able to solve the technical problem. In conclusion, we developed a unique product with new features that cannot be found in other products, such as a spherical shape, a higher frame rate, and a high resolution that can be used for both images and video.

As a result of this study, a new method may be implemented for wireless capsule endoscopes that can help researchers in the future to add more features to the wireless capsule endoscope for the next research study. Through MATLAB code, users and practitioners can also compare different camera calibration methods to remove lens distortion for wireless capsule endoscope camera calibration and white balancing implemented for users and practitioners. Researchers can reconstruct depth images from image FPS, which improves image accuracy and removes wireless capsule endoscope noise. A spherical shape can also pass through a small size, which increases the possibility of including children and patients with swallowing difficulties. The present study provides a wireless capsule endoscope for patients with an examination and diagnosis at a lower cost than others.

## Figures and Tables

**Table 1:** Comparison between our capsule and others.

Capsule	PillCam SB3 Given Imaging	EndoCapsule Olympus America	Mir Liam Intermedia Company	MOM Jianshan and Technology	This Paper’s Capsule
Size	Length: 26.2 mm Diameter: 11.4 mm	Length: 26 mm Diameter: 11 mm	Length: 24.5 mm Diameter: 10.8 mm	Length: 27.9 mm Diameter: 13 mm	Inner diameter: 13.5 mm
Battery life	8 h or longer	8 h or longer	11 h or longer	6–8 h or longer	2 h for full frame or 8 h for 32 fps images
Resolution	340 × 340	512 × 512	320 × 320	640 × 480	400 × 400
Frames per second	2 fps	2 fps	3 fps	2 fps	8–32 fps
Field of view	156°	145°	170°	140°	120°
Communication	Radiofrequency communication	Radiofrequency communication	Human body communication	Radiofrequency communication	Wired

## Data Availability

All data are available in the manuscript.
